# The economic burden of pediatric growth hormone deficiency in Italy: a cost of illness study

**DOI:** 10.1007/s40618-023-02277-z

**Published:** 2024-01-10

**Authors:** M. Cappa, G. Pozzobon, M. Orso, M. Maghnie, G. Patti, F. Spandonaro, S. Granato, G. Novelli, D. La Torre, M. Salerno, B. Polistena

**Affiliations:** 1 Research Area for Innovative Therapies in Endocrinopathies, Bambino Gesù Children’s Hospital, IRCCS, Rome, Italy; 2 IRCCS San Raffaele Hospital, Pediatric Unit—Università Vita-Salute, Milan, Italy; 3 C.R.E.A. Sanità (Centre for Applied Economic Research in Healthcare), Rome, Italy; 4 Department of Pediatrics, IRCCS Istituto Giannina Gaslini, Genoa, Italy; 5 Department of Neuroscience, Rehabilitation, Ophtalmology, Genetics, Maternal and Child Health, University of Genoa, Genoa, Italy; 6 University of Rome Tor Vergata, Rome, Italy; 7 Medical Department, Pfizer Italia, Rome, Italy; 8 Health and Value, Pfizer Italia, Rome, Italy; 9 Global Medical Affairs, Pfizer Rare Disease, Rome, Italy; 10 Department of Translational Medical Sciences, Paediatric Endocrinology Unit, University of Naples ‘Federico II’, Naples, Italy

**Keywords:** Growth hormone deficiency, Children, Cost of illness, Economic burden, Italy

## Abstract

**Purpose:**

Growth hormone deficiency (GHD) is a rare condition with a worldwide prevalence of 1 patient in 4000 to 10,000 live births, placing a significant economic burden on healthcare systems. The aim of this study is to generate evidence on the economic burden of children and adolescents with GHD treated with rhGH and their parents in Italy.

**Methods:**

A cost of illness analysis, adopting the prevalence approach, has been developed, producing evidence on the total annual cost sustained by the Italian National Health System (NHS) and by the society. The study is based on original data collected from a survey conducted among Italian children and adolescents with GHD and their parents.

**Results:**

143 children/adolescents with GHD and their parents participated to the survey, conducted from May to October 2021. Patients had a mean age of 12.2 years (SD: 3.1) and were mostly males (68.5%).

The average direct healthcare cost sustained by the NHS was € 8,497.2 per patient/year; adding the out-of-pocket expenses (co-payments and expenses for private healthcare service), the total expense was € 8,568.6. The indirect costs, assessed with the human capital approach, were € 847.9 per patient/year. The total of direct and indirect cost is € 9,345.1 from the NHS perspective, and € 9,416.5 from a social perspective. The total cost incurred by the Italian NHS for children with GHD (range: 5,708–8,354) was estimated in € 48.5–71.0 million, corresponding to 0.04–0.06% of the total Italian public health expense in the year 2020.

**Conclusions:**

The total annual cost for GHD children is close to € 10,000, and is mainly due to the cost of rhGH treatment. This cost is almost entirely sustained by the NHS, with negligible out-of-pocket expenses.

The economic burden on the Italian NHS for the health care of established GHD children is fourfold higher than the prevalence of the disease in the overall Italian population.

**Supplementary Information:**

The online version contains supplementary material available at 10.1007/s40618-023-02277-z.

## Introduction

Pediatric growth hormone deficiency (GHD) is a rare disorder characterized by an insufficient production of growth hormone by the pituitary gland that causes growth failure and impaired musculoskeletal development [1]. GHD may be congenital, acquired or, in most cases, idiopathic [1, 2].

Recombinant human growth hormone (rhGH), also called somatropin, is indicated for the treatment of GHD patients to attain an adult height appropriate for the child’s genetic potential [1, 3].

The Italian Medicines Agency (AIFA) with the Note #39 of 2004 [4] and its subsequent updates until the most recent one in 2021 [5], regulates the prescription of rhGH treatment and identifies the diagnoses for which it is reimbursed by the Italian National Health System (NHS). In the pediatric age, rhGH is reimbursed for the treatment of children with GHD, Turner syndrome, chronic kidney failure (CKF), infants born small for gestational age (SGA), Prader–Willi syndrome (PWS), short stature homeobox-containing gene deficiency (SHOX-D), and Noonan syndrome.

rhGH is a high-cost drug, and in Italy its cost is entirely covered by the NHS. Other healthcare costs related to GHD include hospitalizations, outpatient visits, diagnostic tests, laboratory exams, and rehabilitation; these costs are almost fully reimbursed by the NHS, while the citizens must contribute with a co-payment of modest entity (so-called “ticket”).

GHD is a rare disease with a worldwide prevalence of 1 patient per 4000–10,000 live births [1], while reported data from Italian registries of rhGH users have shown higher prevalence rates. An Italian study [6] based on the rhGH registry of the Piedmont Region estimated that the prevalence of rhGH treatment exposure in patients < 18 years old treated for hypopituitarism or isolated GHD was 9 per 10,000 residents < 18 years in the year 2004. A more recent report based on the Italian National Registry of Growth hormone Therapy (RNAOC) [7] reported that in Italy there were 5,708 children with GHD aged 3–17 years treated with rhGH in the year 2020; based on the Italian resident population in this age group, the prevalence of GHD would be seven children per 10,000.

To our knowledge, there are no published Italian studies investigating the burden of pediatric GHD. Thus, considering the Italian prevalence of GHD children and the high costs related to their treatment, we were interested in estimating the economic burden of pediatric GHD in Italy.

The aim of this paper is to provide a comprehensive cost of illness analysis of pediatric GHD in Italy, from the point of view of the NHS and of the society. This study is part of a project comprising two other studies: (1) a systematic literature review on pediatric rhGH treatment in Italy, having a focus on epidemiology, quality of life, treatment adherence, and economic impact [8]; (2) a survey on quality of life conducted among children and adolescents with GHD and their parents [9].

## Methods

This study was reported according to the Consolidated Health Economic Evaluation Reporting Standards (CHEERS) statement [10, 11]. A filled CHEERS checklist is available in Online Resource 1. A survey was conducted among children and adolescents with GHD and their parents recruited in four Italian centers, in order to estimate the average direct and indirect costs associated with their care. All costs were expressed in euros, currency year 2021. Children and their parents completed the questionnaires autonomously, without the intervention of the clinicians.

This study consisted of online surveys and did not aim to be a clinical trial, to test a new treatment or to change a patient’s clinical management and, therefore, ethics committee approval was not required in accordance with national legislation and institutional requirements.

The study population includes children and adolescents ≤ 18 years old resident in Italy and their parents, with a confirmed diagnosis of GHD and treated with rhGH.

Four physicians, expert in GHD, were involved in this study. They contributed in drafting the research protocol, in building the questionnaire, and to inform the patients of the existence of the questionnaire.

Data were collected between May and October 2021, using questionnaires administered through the CAPI (Computer-Assisted Personal Interview) method. Patients and their caregivers filled out the questionnaires through a web-based platform, and data were provided anonymously by masking the respondents' IP address.

The collected data included: (1) demographic data: age, gender, region of residence, Local Health Unit; (2) disease-specific data: age at diagnosis, age at signs/symptoms onset, medical specialty of the doctor who made the diagnosis, medical specialty of the doctor treating the patient, if the patient is managed by a referral center; (3) information on health resources utilization in the last 12 months: drugs, medical examinations, diagnostic tests, hospitalizations (ordinary and day hospital), outpatient services, rehabilitation, days of study/work lost due to the disease.

The health care resources used (direct cost) were valued with the rates of the Italian national cost nomenclator (Diagnosis Related Groups—DRG, and specialist outpatient care) [12]. All costs were on a national basis, with the exception of the insulin-like growth factor 1 (IGF-1) test which was calculated as the average of the tariffs applied in two Italian regions of Lazio and Veneto. We calculated both the costs sustained by the Italian National Health System (NHS) and the expenses sustained by citizens (out-of-pocket costs, which include co-payments and spending for private healthcare services).

The drug costs (ex-factory price) were calculated multiplying the mean cost per milligram (mg) for three type of rhGH treatments (pre-filled syringe, vial, and easypod™) by an estimated average annual consumption of 344.2 mg per patient. Three patients did not specify the type of rhGH treatment used, thus we calculated a weighted mean cost per mg for them, based on the frequency distribution in our sample of the other three rhGH types. rhGH consumption was estimated considering: children with an average weight of 37.72 kg, calculated using the weight tables by age for males and females with GHD provided by Takeda et al. [13]; an average rhGH dose of 0.025 mg/kg/day, indicated by the clinical experts as the average prescribed dose in their clinical practice, over a period of 1 year (formula: 37.72 kg × 0.025 mg/kg/day × 365 days = 344.2 mg).

Indirect costs were valued using the human capital method. The loss of productivity per day of work lost was valued at € 126.4, calculated as the Italian Gross Domestic Product (GDP) per capita in the year 2021 (€ 27,814) divided for 220 working days per year.

The number of GHD patients in Italy aged ≤ 18 treated with rhGH in the year 2020 was estimated in n = 5,708; this figure was described in a report based on the Italian National Registry of Growth hormone therapy (RNAOC) held by the “Istituto Superiore di Sanità” (the National Institute of Health in Italy) [7]; this number could underestimate the real number of prevalent cases, because the registry covers 18 out of 20 Italian regions. In a second scenario, we considered as prevalent cases the number of patients using rhGH in the year 2020 according to the data provided by IQVIA (*n* = 8,354) (unpublished data); however, this prevalence could overestimate the real prevalence of GHD children, comprising also adult rhGH users or pediatric rhGH users with other diseases/conditions such as small for gestational age (SGA), Prader-Willi syndrome (PWS), Turner syndrome (TS), chronic kidney disease (CKD), and short stature homeobox-containing gene deficiency (SHOX-D). We presented both prevalence scenarios as a range.

### Statistical analysis

Descriptive statistics were presented. Categorical variables were presented as counts and percentages, while continuous variables as means and standard deviations (SD). The STATA/SE 13 software (Stata, College Station, TX, USA) was used for all the analyses. The sample size of this study was determined considering an estimated mean prevalence of 7000 GHD children in Italy, with a confidence level of 95% and a margin of error of 8%.

## Results

### Healthcare resources use

The overall population included in the survey was 143 children/adolescents with GHD and their parents. Patients had a mean age of 12.2 years (SD: 3.1) and were mostly males (68.5%). The mean age at diagnosis was 7.8 years, being lower for females than for males (6.8 vs 8.3 years), while the mean age at onset of signs/symptoms was 5.7 years. Most of respondents were from south/major islands and north west, while the north east and central Italy were under-represented (Table 1).

**Table 1:** Characteristics of study population

	M	F	M + F
Total sample, *n* (%)	98 (68.5)	45 (31.5)	143
Age (years), mean (SD)	12.6 (3.0)	11.2 (3.1)	12.2 (3.1)
Age group (years), *n* (column %)			
0–3	-	1 (2.2)	1 (0.7)
4–6	5 (5.1)	3 (6.7)	8 (5.6)
7–9	9 (9.2)	7 (15.6)	16 (11.2)
10–12	28 (28.6)	20 (44.4)	48 (33.6)
13–15	41 (41.8)	12 (26.7)	53 (37.1)
16–18	15 (15.3)	2 (4.4)	17 (11.9)
Age at diagnosis (years), mean (SD)	8.3 (3.6)	6.8 (3.6)	7.8 (3.7)
Age at first signs and symptoms (years), mean (SD)	5.8 (4.1)	5.4 (3.5)	5.7 (3.9)
Geographical area, *n* (column %)			
North west	45 (45.9)	21 (46.7)	66 (46.2)
North east	–	2 (4.4)	2 (1.4)
Centre	4 (4.1)	–	4 (2.8)
South and major islands	49 (50.0)	22 (48.9)	71 (49.7)

The specialist visits, diagnostic tests, laboratory examinations, hospitalizations, rhGH treatment, and rehabilitation performed by the patients over a period of 12 months, both in absolute number and as mean (standard deviation) per patient are reported in Table 2. The most frequent visits were at endocrinologist/endocrinologist pediatrician, pediatrician, and psychologist, while the diagnostic tests more often performed were X-ray of the hand and wrist and magnetic resonance imaging of the hypothalamic-pituitary region. Patients performed laboratory tests about twice a year. They rarely underwent ordinary hospitalizations (20 total hospitalizations in a year, 9 of which from a single patient), while they were hospitalized as day hospital in an endocrinology ward 1.4 times a year on average. rhGH was administered through a pre-filled syringe in about half of patients, while 27% used vial and 23% used the easypod™ device. Lastly, 13% of patients performed rehabilitation activities.

**Table 2:** Healthcare resources use

	*N*	Mean per patient (SD)
Study population (*n* = 143)		
Specialist visits		
Geneticist	8	0.06 (0.26)
Endocrinologist/endocrinologist pediatrician	292	2.04 (2.78)
Pediatrician	141	0.99 (3.97)
General Practitioner	20	0.14 (0.92)
Psychologist	47	0.33 (2.22)
Other specialist	29	0.20 (0.94)
Diagnostic tests		
Magnetic resonance imaging of the hypothalamic-pituitary region	43	0.30 (0.96)
X-ray of the hand and wrist	107	0.75 (0.80)
X-ray of the knee	8	0.06 (0.23)
Other tests	64	0.45 (1.10)
Laboratory examinations		
Insulin-like Growth Factor 1 (IGF-1) test	266	1.86 (0.35)
Other blood tests	270	1.89 (0.32)
Hospitalizations (ordinary)		
General medicine	–	–
Endocrinology	8	0.06 (0.31)
Pediatrics	2	0.01 (0.17)
Other wards	10	0.07 (0.61)
Day hospital		
General medicine	4	0.03 (0.20)
Endocrinology	201	1.41 (1.08)
Pediatrics	4	0.03 (0.20)
Other wards	4	0.03 (0.20)
Rehabilitation	18	0.13 (0.33)
rhGH treatment, N (% of patients)		
Pre-filled syringe	69 (48.3%)	
Vial	38 (26.6%)	
Easypod™	33 (23.1%)	
Other methods	3 (2.1%)	

### Direct costs

Table 3 shows the unit costs for each of the healthcare resources considered. The higher costs are those of rhGH treatment, of which the pre-filled syringe is the most expensive, and hospitalizations.

**Table 3:** Unit costs

Healthcare service	Tariffs (€)	Source
Specialist visit (any type)	20.66	Code 89.7
Psychological visit	19.37	Code 94.1
Magnetic resonance imaging of the hypothalamic-pituitary region	166.58	Code 88.91.1
X-ray of the hand and wrist	14.20	Code 88.23
X-ray of the knee	21.17	Code 88.23
Rehabilitation	19.10	Code 93.96
Yearly rhGH treatment with pre-filled syringe, mean	8,398.8	Mean cost per mg (€ 24.4) * 344.2 mean mg consumption per year
Yearly rhGH treatment with vial, mean	5,541.9	Mean cost per mg (€ 16.1) * 344.2 mean mg consumption per year
Yearly rhGH treatment with easypod™, mean	6,292.3	Mean cost per mg (€ 18.28) * 344.2 mean mg consumption per year
Yearly rhGH treatment with unspecified method, mean	7,126.9	Weighted mean cost per mg (€ 20.7) * 344.2 mean mg consumption per year
Hospitalization (ordinary)	1,312.1	National tariff related to DRG 301
Day hospital	621.1	National tariff related to DRG 301
Laboratory examinations		
Insulin-like Growth Factor 1 (IGF-1) test	20.0	Mean tariffs of Lazio and Veneto Regions (Italy)
Growth hormone (GH)	10.48	Code 90.35.1
Thyroid-stimulating hormone (TSH)	5.46	Code 90.42.1
Follicle-stimulating hormone (FSH)	6.21	Code 90.23.3
Prolactin	7.20	Code 90.32.3
Estradiol	9.11	Code 90.19.2
Testosterone	9.78	Code 90.41.3
Complete blood count (CBC)	3.17	Code 90.62.2
Glycaemia	1.17	Code 90.27.1
Anti-gliadin antibodies (IgA, IgG)	10.27	Code 90.49.5

Considering the healthcare resources used in the last 12 months (period 2020–2021) and their unit costs, we calculated the mean costs per person/year (Table 4). The mean direct healthcare cost sustained by the NHS was € 8497.2, while the out-of-pocket (co-payment + private healthcare) spending was € 71.4, for a total expenditure of € 8568.6.

**Table 4:** Mean costs (€) per person/year

	Total cost	NHS cost	Out-of-pocket spending	
			Co-payment by patients	Private healthcare
Direct healthcare costs (total)	8568.6	8497.2	8.2	63.2
Drugs	7129.4	7124.0	2.8	2.6
Hospitalizations	1108.6	1108.6	–	–
Laboratory examinations	166.1	154.5	1.4	10.2
Diagnostic tests	69.2	60.4	1.5	7.3
Medical visits	63.4	47.3	2.5	13.6
Rehabilitation	31.9	2.4	–	29.5
Indirect costs (total)	847.9			
Days of study/work lost by patients due to illness, mean	3.1			
Productivity loss of patients	390.8			
Days of study/work lost by caregivers due to illness, mean	3.6			
Productivity loss of caregivers	457.1			
Total costs (direct + indirect; NHS), mean	9345.1			
Total costs (direct + indirect; NHS + out-of-pocket), mean	9416.5			

rhGH treatment represents the 83.2% of the total expenses, while hospitalizations the 12.9%. The out-of-pocket expenses was marginal compared to the NHS cost (0.8% of the total expenses).

### Indirect costs

As for indirect costs, each patient has lost on average 3.1 days of study/work, and their caregivers 3.6 days, with an average annual indirect cost of € 847.9.

### Cost of illness

Total cost sustained by NHS results in € 9345.1 per patient, that raise to € 9416.5 considering also the out-of-pocket expenses.

Based on the total direct per patient cost sustained by the NHS and the GHD prevalence, considering the prevalent GHD patients < 18 years old reported by the Italian National Registry of Growth hormone therapy for the year 2020 (*n* = 5,708 prevalent cases), the annual cost of pediatric GHD would be € 48.5 million; instead, considering the rhGH users in the year 2020 according to the data provided by IQVIA (*n* = 8,354 rhGH users), the total cost would be € 71 million. These two estimated total costs, compared to the Italian public health expenditure in the year 2020 (€ 123,474 million), correspond to 0.04% and 0.06%, compared to a prevalence of GHD children in the overall Italian population of 0.01%, that is fourfold higher than the prevalence.

## Discussion

The aim of this study was to quantify the pediatric GHD cost of illness in Italy. Our results show that the mean direct healthcare cost sustained by the NHS was € 8497.2 per patient/year; considering also private spending (out-of-pocket expenses), the total cost was € 8568.6. Indirect costs, that were calculated valuing the days of study/work lost by patients and caregivers, account for € 847.9 per patient/year. The sum of direct and indirect cost is € 9345.1 from the NHS perspective, and € 9416.5 from a societal perspective. The projection of these costs on the estimated prevalent cases of Italian GHD children (range: 5708–8354) indicates a range of total cost of € 48.5–71 million, corresponding to 0.04–0.06% of the total Italian public health expenses in the year 2020. Another Italian study [14] reported an estimated prevalence of GHD patients < 18 years treated with rhGH of 5,157 subjects in the year 2012, that is lower than that reported by the Italian rhGH user registry [7] for the year 2020 (*n* = 5,708); considering this prevalence, according to our estimated annual cost per patient sustained by the NHS, the total cost for these patients would be € 43.8 million.

The total expenses for GHD children out of the total Italian health expenses is therefore fourfold higher than the prevalence of pediatric GHD out of the overall Italian population (0.04–0.06% vs 0.01%). Expenditure higher than the prevalence of the disease is common in the case of rare diseases. However, this can be considered acceptable as the treatment of GHD lasts a limited number of years and avoids further costs during the rest of the patients' lives.

To our knowledge, this is the first Italian cost of illness study on pediatric GHD patients, thus we were not able to compare our estimates with those of other similar Italian studies.

A recent systematic review [8] identified two Italian studies addressing the economic impact of rhGH treatment in GHD children.

The study by Foo et al. [15] described a cost-consequence analysis that compared the easypod ™ device with other devices in the treatment of GHD children in Italy. In this study, only the total costs of the different devices for a complete multi-year rhGH treatment (until the achievement of the final height) were reported, not describing the annual cost per patient; in addition, no other healthcare costs were described, and therefore this study cannot be compared with our study.

Another Italian study by Pagani et al. [16] aimed to assess the cost-effectiveness of rhGH treatment in two groups of subjects: 12 children without GH deficiency (bioinactive GH) and 12 GHD children. The authors did not find significant differences in the mean annual costs for rhGH treatment between GHD children (€ 12,348 ± 2,018) and subjects with reduced GH biological activity (€ 11,355 ± 1,748). The annual costs of rhGH treatment reported in this study were higher than ours (range: + 59%, + 73%).

A study from the United States [17] based on claims data aimed to estimate the health care costs among children with GHD who had either Medicaid (*n* = 6820) or commercial health insurance (*n* = 14,070). This study reported an average unadjusted annual GHD-related costs of $ 18,069 (somatropin outpatient pharmacy costs: $ 11,951; inpatient costs: $ 5471; outpatient costs: $ 647) for Medicaid-insured patients and $27,893 (somatropin outpatient pharmacy costs: $ 24,130; inpatient costs: $ 1934; outpatient costs: $ 1829) for commercially insured patients. Converted in euros (currency year 2021), the Medicaid-insured patients total cost was € 13,664, while the commercially insured patients total cost was € 21,094. Compared to the total direct healthcare costs estimated in the present study, both US estimates were higher, + 59% and + 146%, respectively. In addition, the authors stated that the costs of expensive drugs such as somatropin may be overestimated using claims data, particularly for commercial plans where there is less cost transparency and negotiated prices may vary highly between plans. Furthermore, considering the differences between the Italian and the US healthcare systems, the results of these two studies are not directly comparable.

This study is part of a wider project including a systematic literature review [8] and a quality of life study for GHD children in Italy [9]. The systematic review [8] sought to describe the epidemiology of growth hormone treatment-indicated diseases in Italy, the adherence to rhGH treatment and the factors linked to non-adherence, the economic impact of rhGH treatment in Italy, and the quality of life of patients treated with rhGH and their caregivers in Italy. The quality-of-life study [9] was based on the same cohort of patients and their parents as the present economic study. This study showed that generic health-related quality of life, measured by the EQ-5D questionnaire, in GHD-treated patients was high and similar to that of healthy people. Disease-specific quality of life assessed by the QoLISSY questionnaire was also good and comparable to that of international reference values of GHD/idiopathic short stature patients.

The present economic analysis is based on a survey that includes an adequate sample size (*n* = 143 children and their parents) to provide reliable estimates (confidence level 95%, margin of error 8%). We considered both direct and indirect costs, adopting both the NHS and societal perspectives.

Potential limits of this study are that subjects from north east and central Italy are under-represented compared to the Italian population, limiting the representativeness of the sample.

Another potential limitation is that we did not collect data on survey non-responders, so we could not calculate the response rate. Therefore, we were not able to compare the characteristics of responders and non-responders, in order to assess the potential non-response bias.

In addition, we did not consider other potential direct non-medical costs, such as transport costs of the patients and caregivers to reach health facilities, within or outside the region of residence.

## Conclusion

The total annual cost per GHD children reaches almost 10,000 euros, mostly due to the drug cost. This cost is almost fully sustained by the NHS, with modest out-of-pocket costs by the household. The Italian prevalent population of GHD children in treatment with rhGH was estimated to range from 5708 to 8354 subjects, equal to the 0.01% of the Italian population. The total economic burden for the Italian NHS results in € 48.5–71 million, corresponding to the 0.04–0.06% of the total Italian public health expenses in 2020. Compared to the pediatric GHD prevalence, the incidence on public health expenditure is fourfold higher.

### Supplementary Information

Below is the link to the electronic supplementary material.

Supplementary file1 (PDF 119 KB)

## Data Availability

The datasets generated during and/or analyzed during the current study are available from the corresponding author on reasonable request.
