# Supplementary material for: The economic burden of pediatric growth hormone deficiency in Italy: a cost of illness study
Source: J Endocrinol Invest. 2024 Jan 10;47(7):1743–50. doi: 10.1007/s40618-023-02277-z (PMC11196344; doi:10.1007/s40618-023-02277-z)
Supplement: Supplementary file 1 — Supplementary file1 (PDF 119 KB) [file 40618_2023_2277_MOESM1_ESM.pdf]

CHEERS 2022 Checklist

Topic	No.	Item	Location where item is reported
**Title**			
	1	Identify the study as an economic evaluation and specify the interventions being compared.	Title page, Lines 2-3
**Abstract**			
	2	Provide a structured summary that highlights context, key methods, results, and alternative analyses.	Abstract, Lines 35-60
**Introduction**			
**Background and objectives**	3	Give the context for the study, the study question, and its practical relevance for decision making in policy or practice.	Introduction, Lines 65-97
**Methods**			
**Health economic analysis plan**	4	Indicate whether a health economic analysis plan was developed and where available.	Not applicable
**Study population**	5	Describe characteristics of the study population (such as age range, demographics, socioeconomic, or clinical characteristics).	Methods, Lines 109-110
**Setting and location**	6	Provide relevant contextual information that may influence findings.	Methods, Lines 101-102; 113-114
**Comparators**	7	Describe the interventions or strategies being compared and why chosen.	Not applicable
**Perspective**	8	State the perspective(s) adopted by the study and why chosen.	Methods, Lines 126-128
**Time horizon**	9	State the time horizon for the study and why appropriate.	Not applicable
**Discount rate**	10	Report the discount rate(s) and reason chosen.	Not applicable
**Selection of outcomes**	11	Describe what outcomes were used as the measure(s) of benefit(s) and harm(s).	Not applicable
**Measurement of outcomes**	12	Describe how outcomes used to capture benefit(s) and harm(s) were measured.	Not applicable
**Valuation of outcomes**	13	Describe the population and methods used to measure and value outcomes.	Not applicable
**Measurement and valuation of resources and costs**	14	Describe how costs were valued.	Methods, Lines 123-126; 129-137
**Currency, price date, and conversion**	15	Report the dates of the estimated resource quantities and unit costs, plus the currency and year of conversion.	Methods, Lines 103-104

Topic	No.	Item	Location where item is reported
**Rationale and description of model**	16	If modelling is used, describe in detail and why used. Report if the model is publicly available and where it can be accessed.	Not applicable
**Analytics and assumptions**	17	Describe any methods for analysing or statistically transforming data, any extrapolation methods, and approaches for validating any model used.	Methods , Lines 153-157
**Characterising heterogeneity**	18	Describe any methods used for estimating how the results of the study vary for subgroups.	Not applicable
**Characterising distributional effects**	19	Describe how impacts are distributed across different individuals or adjustments made to reflect priority populations.	Not applicable
**Characterising uncertainty**	20	Describe methods to characterise any sources of uncertainty in the analysis.	Not applicable
**Approach to engagement with patients and others affected by the study**	21	Describe any approaches to engage patients or service recipients, the general public, communities, or stakeholders (such as clinicians or payers) in the design of the study.	Not applicable
**Results**			
**Study parameters**	22	Report all analytic inputs (such as values, ranges, references) including uncertainty or distributional assumptions.	Not applicable
**Summary of main results**	23	Report the mean values for the main categories of costs and outcomes of interest and summarise them in the most appropriate overall measure.	Results, 181-204
**Effect of uncertainty**	24	Describe how uncertainty about analytic judgments, inputs, or projections affect findings. Report the effect of choice of discount rate and time horizon, if applicable.	Not applicable
**Effect of engagement with patients and others affected by the study**	25	Report on any difference patient/service recipient, general public, community, or stakeholder involvement made to the approach or findings of the study	Not applicable
**Discussion**			
**Study findings, limitations, generalisability, and current knowledge**	26	Report key findings, limitations, ethical or equity considerations not captured, and how these could affect patients, policy, or practice.	Discussion, Lines 208-270
**Other relevant information**			
**Source of funding**	27	Describe how the study was funded and any role of the funder in the identification, design, conduct, and reporting of the analysis	281-283
**Conflicts of interest**	28	Report authors conflicts of interest according to journal or International Committee of Medical Journal Editors requirements.	284-304

From: Husereau D, Drummond M, Augustovski F, et al. Consolidated Health Economic Evaluation Reporting Standards 2022 (CHEERS 2022) Explanation and Elaboration: A Report of the ISPOR CHEERS II Good Practices Task Force. Value Health 2022;25. doi:10.1016/j.jval.2021.10.008
